# Multichannel, Portable and Low-Cost Source Measure Unit Platform for the Continuous Characterization of Organic Electrochemical Transistor Biosensors

**DOI:** 10.3390/s26206526

**Published:** 2026-10-15

**Authors:** Michele Campanini, Lorenzo Vigna, Davide Vurro, Pasquale D’Angelo, Martina Cicolini, Simone Luigi Marasso, Matteo Cocuzza, Alberto Ballesio

**Affiliations:** 1 Department of Applied Science and Technology (DISAT), Politecnico di Torino, Corso Duca Degli Abruzzi 24, 10129 Turin, Italy; michele.campanini@polito.it (M.C.); martina.cicolini@polito.it (M.C.); simone.marasso@polito.it (S.L.M.); matteo.cocuzza@polito.it (M.C.); alberto.ballesio@polito.it (A.B.); 2 PiQuET LAB, Piemonte Quantum Enabling Technology, Str. delle Cacce, 91, 10135 Turin, Italy; 3 Institute of Materials for Electronics and Magnetism, IMEM-CNR, Parco Area delle Scienze 37/A, 43124 Parma, Italy; davide.vurro@cnr.it (D.V.); pasquale.dangelo@cnr.it (P.D.)

**Keywords:** Source Measure Unit (SMU), Organic Electrochemical Transistor (OECT), in vitro platform, continuous monitoring, Arduino prototyping

## Abstract

Organic Electrochemical Transistors (OECTs) have emerged as continuous monitoring sensors for in vitro models. Source Measure Units (SMUs) are typical instruments used to simultaneously control input and output signals in OECTs. Conventional SMUs are unsuitable for in vitro applications due to their limited channel count, large footprint and high cost. This work presents the design and development of a multichannel, portable and cost-effective SMU platform for the continuous and long-term characterization of OECT biosensors. The hardware is based on a breadboard interfacing via custom connections to a six-OECT setup integrated into a standard culture plate and operated within a cell incubator. System functionality is managed by an Arduino Nano microcontroller (Arduino, Monza, Italy) through custom-developed software, enabling automated characterization of the OECTs. Platform performance was evaluated by comparing OECT I–V characteristics and time responses against the commercial B2912A SMU (Keysight Technologies, Santa Rosa, CA, USA), using phosphate-buffered saline (PBS) to mimic biological samples. Long-term continuous operation was verified through a 5 day recording session with a 30 min sampling interval. Experimental results demonstrated consistent functionality with satisfactory accuracy for the targeted application. The proposed platform is a promising alternative to conventional SMUs for the continuous and long-term monitoring of OECT biosensors. Future work will focus on hardware optimization and full biological validation through OECT-based monitoring of in vitro models.

## 1. Introduction

In the field of biosensing technologies, Organic Electrochemical Transistors (OECTs) represent a novel category of biosensors. These devices consist of gate, drain, and source electrodes, along with an ion-permeable organic semiconducting (OSC) channel [1]. OECTs operate in contact with an electrolyte, where gate–source voltage variations (input) modulate the drain–source current (output) through ion injection into the channel [2]. To date, OECTs have been exploited to detect a wide range of analytes, including enzymes, metabolites, antibodies and nucleic acids [3]. Thanks to their OSC properties, they have also been used to monitor biological models. Indeed, the most common channel material, poly(3,4-ethylenedioxythiophene):polystyrene sulfonate (PEDOT:PSS), exhibits electrochemical stability and biocompatibility and enables low-voltage operation (<1 V), conferring high signal amplification to OECTs [1,2,4].

OECTs are typically characterized using commercial Source Measure Units (SMUs), instruments that simultaneously supply and measure voltage and current on the same channel [5]. In OECT biosensor characterization, the drain–source current (I_ds_) is generally measured while supplying a variable gate–source voltage (V_gs_) and a constant drain–source voltage (V_ds_). Through this approach, biochemical processes are examined via the resulting transfer characteristics and time responses [6]. Common SMUs used in research include the B2900 Series (Keysight Technologies, Santa Rosa, CA, USA) and the 2400 Standard Series (Keithley Instruments, Cleveland, OH, USA) [7,8,9]. As a representative model, the Keysight B2912A features full four-quadrant operation, a maximum range of ±210 V/±3 A, a maximum resolution of 100 nV/10 fA, a noise level below 100 nV/10 fA, a maximum sampling rate of 10 μs, and a memory buffer of 100,000 data points. It allows the user to configure versatile triggers and current and voltage ranges and generate customized waveforms through GUI software and SCPI programming. It supports USB, LAN, and GPIB communication with a PC and wiring via BNC connectors. Even though commercial SMUs are state-of-the-art equipment, they are not intended for in vitro experimentation. Standard models provide only one or two channels, which are insufficient for biological replicates. Modular and high-channel-count models are significantly more expensive, constituting a considerable barrier for research institutions. Another constraint is their bulkiness, which complicates transportation and setup in biological laboratories [7,10]. Regarding software, the limited memory of standard models is inadequate for long-term measurements, necessitating a PC for data logging. The specialized syntax of the SCPI (Standard Commands for Programmable Instruments) language [11] requires dedicated study for proper coding.

Hence, there is a need for a compact, affordable and user-friendly SMU that is compatible with in vitro biosensing. Many low-cost prototypes, although not specifically designed for biosensors, have been developed to date. They are composed of Arduino microcontrollers [5,7,8,12,13] or NI PXI embedded controllers [14], digital-to-analog converters (DACs), analog-to-digital converters (ADCs), power supplies, analog circuits to interface with the device under test (DUT) and supporting hardware for data transmission and processing. Functionalities are controlled by custom software written in various programming languages. With regard to biosensing, three recently proposed prototypes are particularly noteworthy. The first, developed by Galanti and coworkers [9], integrates a commercial power supply, a general-purpose CPU, and an analog circuit for the characterization of graphene field-effect transistors (FETs). It features full four-quadrant operation and a high signal-to-noise ratio and is equipped with an electromagnetic interference (EMI) shielding box. The second, constructed by Bryantono et al. [15], is an STM32F1-based circuit for I–V tracing of FET biosensors. It features an optimized architecture to minimize parasitic elements, an enclosure for heat dissipation and a LabVIEW GUI for data analysis. The third, reported by Park and colleagues [10], is a WeMos D1-controlled web-based system for measuring the transfer characteristics of ionic liquid-gated FETs. It can automatically store real-time measurements on a cloud server via wireless communication, generating large datasets for long-term experiments.

Despite these achievements, these three systems lack a biosensor integration setup (thereby necessitating external probes), support only single-device testing, are limited to I–V characterization (no time response or temporal measurements are reported), and require additional hardware and software resources for data analysis. Based on the aforementioned limitations, in this work we have developed a multichannel, portable and low-cost SMU platform for the I–V and time response characterization of OECT biosensors. The platform controls a customized six-OECT setup integrated into a standard culture plate and operated within a cell incubator. The sensorized plate communicates with the breadboard via a dedicated wiring setup tailored for in vitro procedures. The circuitry is based on an Arduino Nano microcontroller unit (MCU) and easy-to-use components, enabling automatic measurement and data logging. The breadboard-based implementation facilitates prototyping while maintaining an acceptable offset (≈40 µA) and noise level (≈180 nA) for various OECT biosensing applications. The user-friendly Arduino IDE is used for program development and uploading to the MCU. This system is capable of continuously and autonomously monitoring six OECT biosensors over long-term periods, yielding a large dataset. Table 1 and Table 2 compare the proposed system with previously reported works, focusing on key features and main electrical specifications, respectively. The present SMU offers more measurement methods and channels (which account for its higher cost) and requires only a power supply as supporting hardware. Unlike most of the cited works, the developed software is open-source and accessible in Supplementary Materials. Since each system is tailored to a particular application, its electrical specifications exhibit substantial variability. The proposed platform is customized for OECTs, featuring a per-channel range of 5 V/12 mA, which fully encompasses the OECT operating domain, and a resolution of 1.22 mV/78 nA, thereby enabling the characterization of a broad range of OECT biosensors.

In this paper, we present the developed SMU platform and validate its OECT characterization performance against the commercial Keysight B2912A SMU. Results demonstrate that the proposed system delivers reliable performance and satisfactory accuracy, providing a cost-effective alternative to commercial SMUs for the long-term, continuous monitoring of OECT biosensors. Future work and ongoing developments focus on hardware optimization and biological validation through the observation of OECT-based cell models.

## 2. Materials and Methods

### 2.1. Hardware Design

#### 2.1.1. SMU Circuit

**Figure 1:**
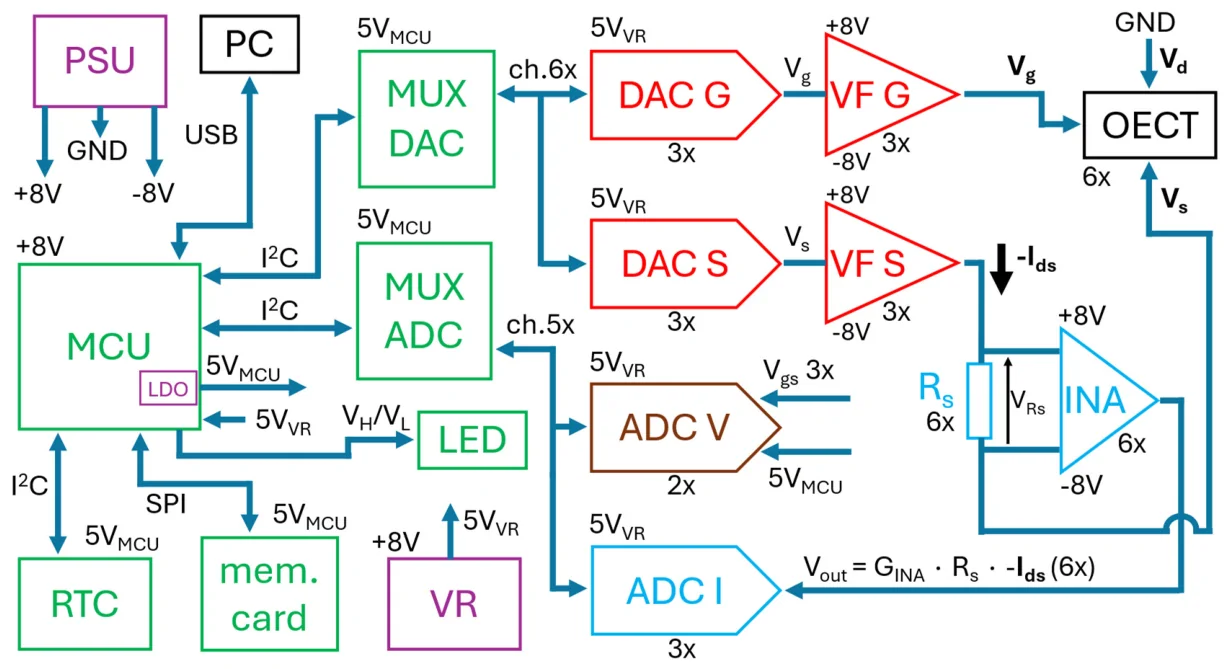
Block diagram of the developed SMU circuit, composed of 5 functional subsystems: digital (green), voltage-driving (red), voltage-sensing (brown), current-sensing (blue) and power supply (purple).

The proposed SMU circuit, whose block diagram is shown in Figure 1, comprises five functional subsystems: digital, voltage-driving, voltage-sensing, current-sensing and power supply. The digital subsystem includes an MCU, a real-time clock (RTC) for timestamping, a memory card for data and timestamp storage, a red light-emitting diode (LED) for signaling the acquisition phase, and two multiplexers (MUXs) for converter selection. The MCU communicates with the RTC and MUXs via the Inter-Integrated Circuit (I^2^C) protocol and with the memory card via the Serial Peripheral Interface (SPI) protocol. Although not essential for operation, a PC can be connected to the MCU via USB for real-time checks. The voltage-driving subsystem consists of six DACs, three generating the gate voltages (V_g_) and three driving the source voltages (V_s_) for the OECTs. These voltages feed six operational amplifiers (op-amps) configured as voltage followers (VFs), three driving the V_g_ and three driving the V_s_ signals, respectively. Each VF controls two parallel OECTs by supplying the required current and a load-independent driving voltage. Thus, OECT pairs share the same driving voltage, whereas their currents are acquired independently, as explained below. The drain voltages (V_d_) of the OECTs are tied to GND (0 V). The voltage-sensing subsystem is composed of two ADCs that measure the three gate–source voltages (V_gs_) and the 5 V power supply, as explained below. The current-sensing subsystem is based on six high-side shunt resistors (R_s_) in series with the corresponding OECTs, six instrumentation amplifiers (INAs), and three ADCs. The drain–source currents (I_ds_) of the OECTs are acquired using the shunt-resistance method: I_ds_, driven by V_ds_ and flowing through R_s_, produces a proportional voltage drop according to Ohm’s law (V_Rs_ = I_ds_∙R_s_), which is amplified by the INA (V_out_ = G_INA_∙V_Rs_, where G_INA_ is the INA gain) and measured by the ADC. Accordingly, I_ds_ is computed as follows:

I_ds_ = −V_out_/(G_INA_∙R_s_). (1)

The power supply subsystem includes a power supply unit (PSU), providing a common GND, single +8 V, and bipolar ±8 V rails, a voltage regulator (VR) generating a single 5 V supply (5 V_VR_), and the low-dropout regulator (LDO) integrated into the MCU, providing a single 5 V supply (5 V_MCU_). The MCU and VR are powered by +8 V, the op-amps by ±8 V, the converters by 5 V_VR_, and the digital subsystem by 5 V_MCU_. The two 5 V sources are used to distribute the power load and prevent interference between analog and digital circuits. These supply rails are inspected by the MCU and the voltage-sensing ADC, respectively, to detect potential power anomalies.

The following sections describe the circuit components and the subsystem schematics designed using KiCad (v9.0.5). Recurring components are omitted to avoid redundancy.

#### 2.1.2. Digital Subsystem

**Figure 2:**
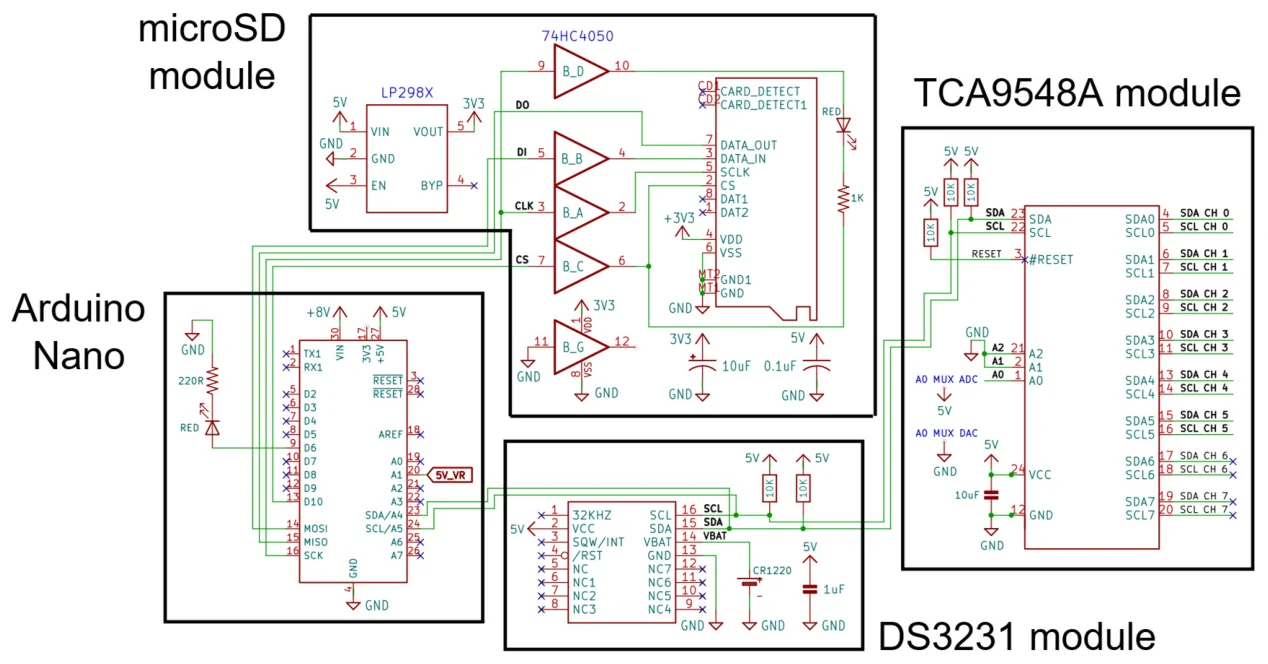
Electronic schematic of the digital subsystem.

The digital subsystem (Figure 2) implements Arduino Nano (Arduino) as the MCU, which is a compact, low-power and user-friendly development board based on the ATmega328 microcontroller (Microchip Technology, Chandler, AZ, USA) [13]. The other devices are based on breakout boards (hereafter referred to as modules), which are modular printed circuit boards (PCBs) hosting the integrated circuit (IC) and auxiliary components, allowing ICs to be easily managed through breadboard-friendly pins [16]. The RTC consists of the DS3231 module (Maxim Integrated, San Jose, CA, USA; Adafruit Industries, New York, NY, USA), a battery-integrated, highly accurate clock capable of maintaining date and time information for long periods. MUXs are based on the TCA9548A module (Texas Instruments, Dallas, TX, USA; Adafruit Industries), a low-standby-current I^2^C switch capable of selecting among up to eight devices. The high-speed 8 GB USD500S microSDHC (Transcend Information, Taipei, Taiwan) is used as a memory card integrated into the microSD card adapter module (Adafruit Industries).

#### 2.1.3. Voltage-Driving Subsystem

**Figure 3:**
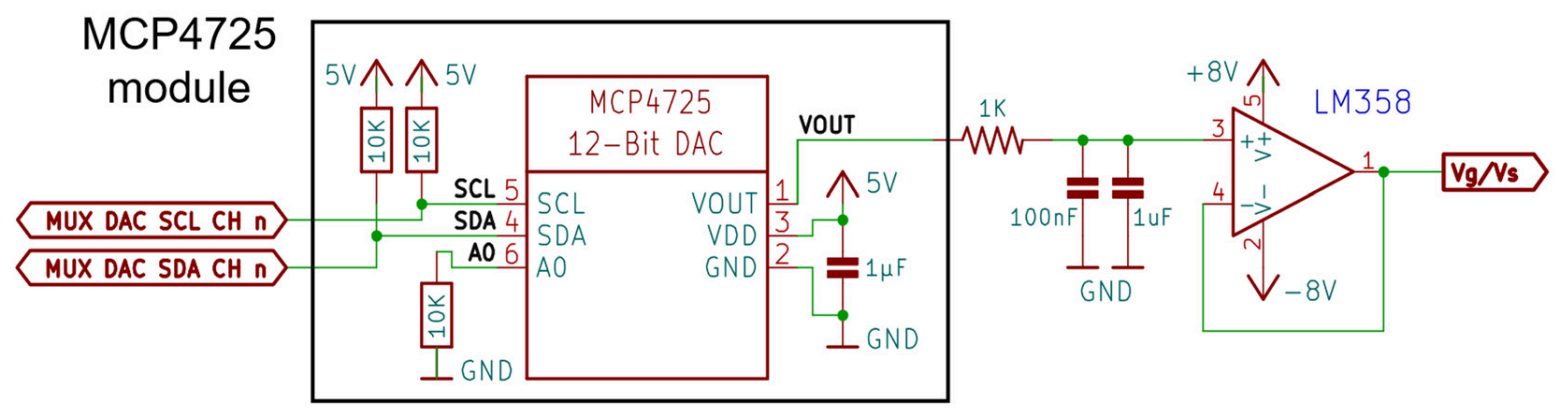
Electronic schematic of the voltage-driving subsystem. Recurring components are omitted for simplicity.

The voltage-driving subsystem (Figure 3) utilizes the rail-to-rail, low-power 12-bit MCP4725 modules (Microchip Technology; Adafruit Industries) as DACs. The I^2^C pins of each module are connected to the corresponding I^2^C channel pins of the DAC-selecting MUX. The module output signal is processed by a low-pass RC filter consisting of a 1 kΩ resistor (CFR25 type) in parallel with a 1 µF ceramic capacitor and a 100 nF film capacitor. This filter, with a cutoff frequency of ≈145 Hz, attenuates a broad range of noise frequencies while preserving the working signals. VFs are implemented with the LM358 IC (Texas Instruments), which is a cost-effective, low-offset, and unity-gain-stable dual op-amp.

#### 2.1.4. Voltage and Current-Sensing Subsystems

**Figure 4:**
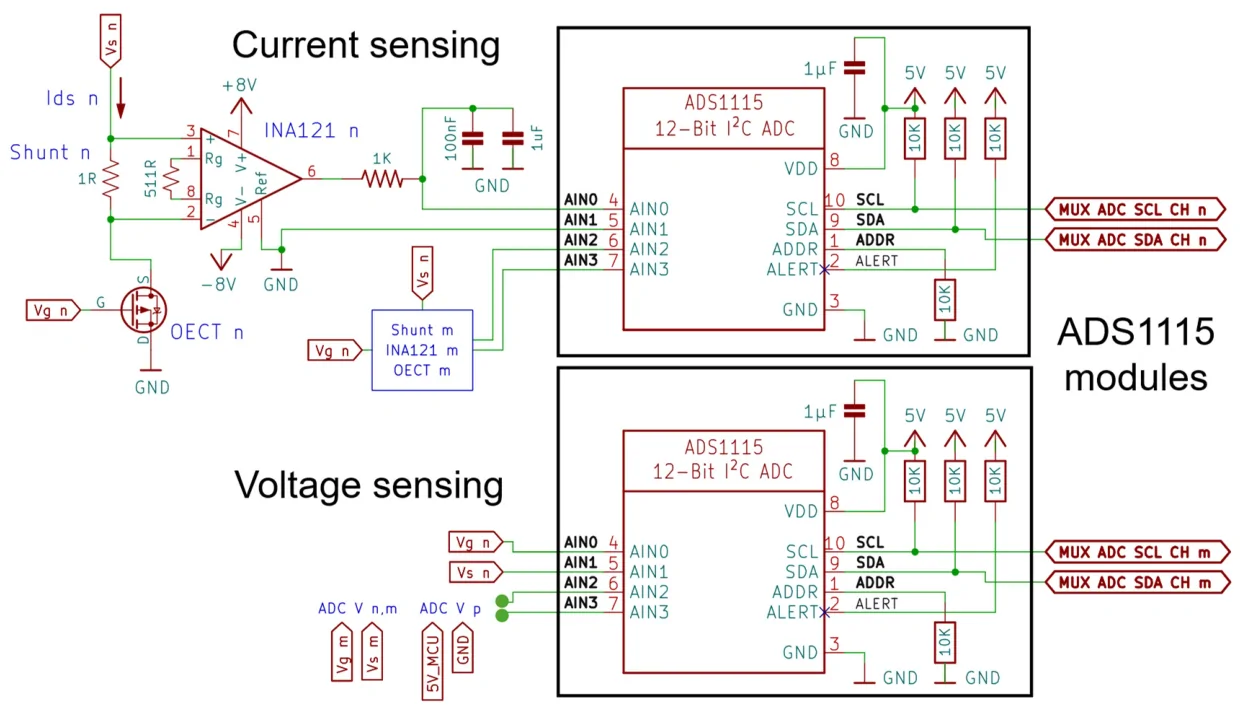
Electronic schematic of the voltage and current-sensing subsystems. Recurring components are omitted for simplicity.

The ADCs of the sensing subsystem (Figure 4) are based on the four-channel, low-power 16-bit ADS1115 module (Texas Instruments; Zhiwei Robotics, Shanghai, China), configured in differential mode for common noise reduction. By incorporating a low-drift voltage reference and a programmable gain amplifier (PGA), ADS1115 supports six input full-scale ranges (FSRs) from ±0.256 to ±6.144 V, with corresponding resolutions from 7.812 to 187.5 µV. It offers eight programmable data rate settings, ranging from 8 samples per second (SPS) up to 860 SPS, clocked by an integrated oscillator. The I^2^C pins are connected to the corresponding I^2^C channel pins of the ADC-selecting MUX. Shunt resistors consist of precision 1 Ω resistors (E96 series), whose ends are connected to the positive and negative input pins of the INA121 instrumentation amplifiers (Texas Instruments). This high-accuracy INA exhibits high common-mode rejection, low voltage offset and drift, and low bias and quiescent currents. By placing an external resistor R_G_ of 511 Ω (E96 series) between its two R_g_ pins, the INA gain (G_INA_ = V_out_/(V_in+_ − V_in−_)) was set to 100, according to

G_INA_ = 1 + 50 kΩ/R_G_. (2)

V_out_ is subsequently processed by a low-pass filter identical to that of the voltage-driving subsystem.

#### 2.1.5. Power Supply Subsystem

**Figure 5:**
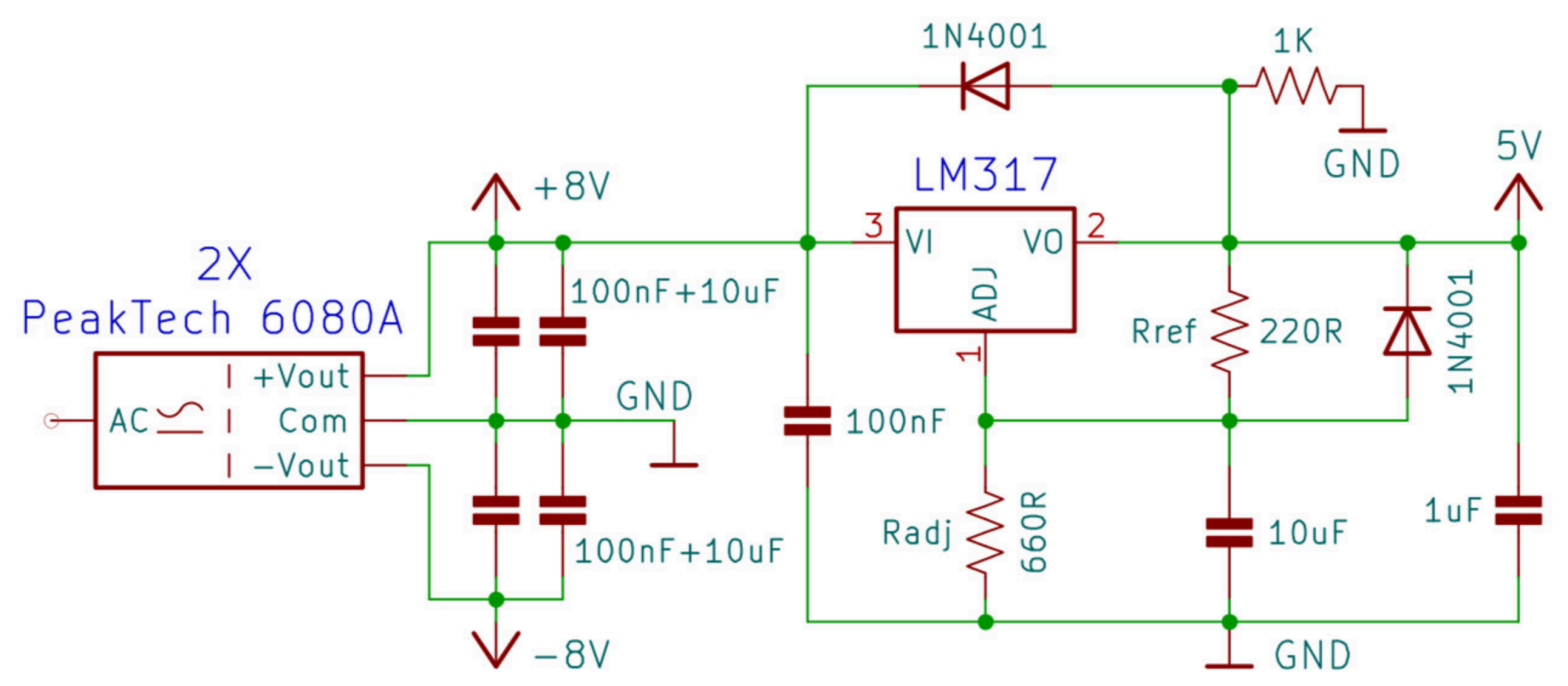
Electronic schematic of the power supply subsystem.

The series connection of two 6080A linear-regulated power supplies (PeakTech, Ahrensburg, Germany) is used as the bipolar PSU, as shown in the subsystem schematic in Figure 5. A capacitor-input filter placed on the ±8 V lines, consisting of a 100 nF film capacitor in parallel with a 10 µF tantalum capacitor, suppresses high-frequency noise on the power rails. The LM317 VR (Texas Instruments) is placed downstream of the PSU to generate 5 V_VR_ from +8 V. This adjustable three-pin regulator supplies an output voltage equal to V_out_ = V_ref_ ∙ (1 + R_adj_/R_ref_) + I_adj_ ∙ R_adj_, where V_ref_ = 1.25 V, I_adj_ ≈ 50 µA, R_ref_ is set to 220 Ω [17], and R_adj_ is set to 660 Ω to obtain V_out_ = 5 V. The PSU output was set to 8 V to guarantee a minimum headroom of 3 V on the VR, and a 1 kΩ load resistor was placed at the VO pin to achieve a minimum output current of 5 mA, as recommended in the component datasheet. Moreover, LM317 is supported by the following external circuitry: a 100 nF bypass film capacitor at the VI pin (for input voltage stability), a 10 µF tantalum capacitor at the ADJ pin (for ripple rejection improvement), a 1 µF ceramic capacitor at the VO pin (for transient response improvement), and two protective 1N4001G diodes between the VO and VI pins and the ADJ and VO pins, respectively (to prevent capacitors from discharging across V_out_).

#### 2.1.6. OECT and Wiring Setup

**Figure 6:**
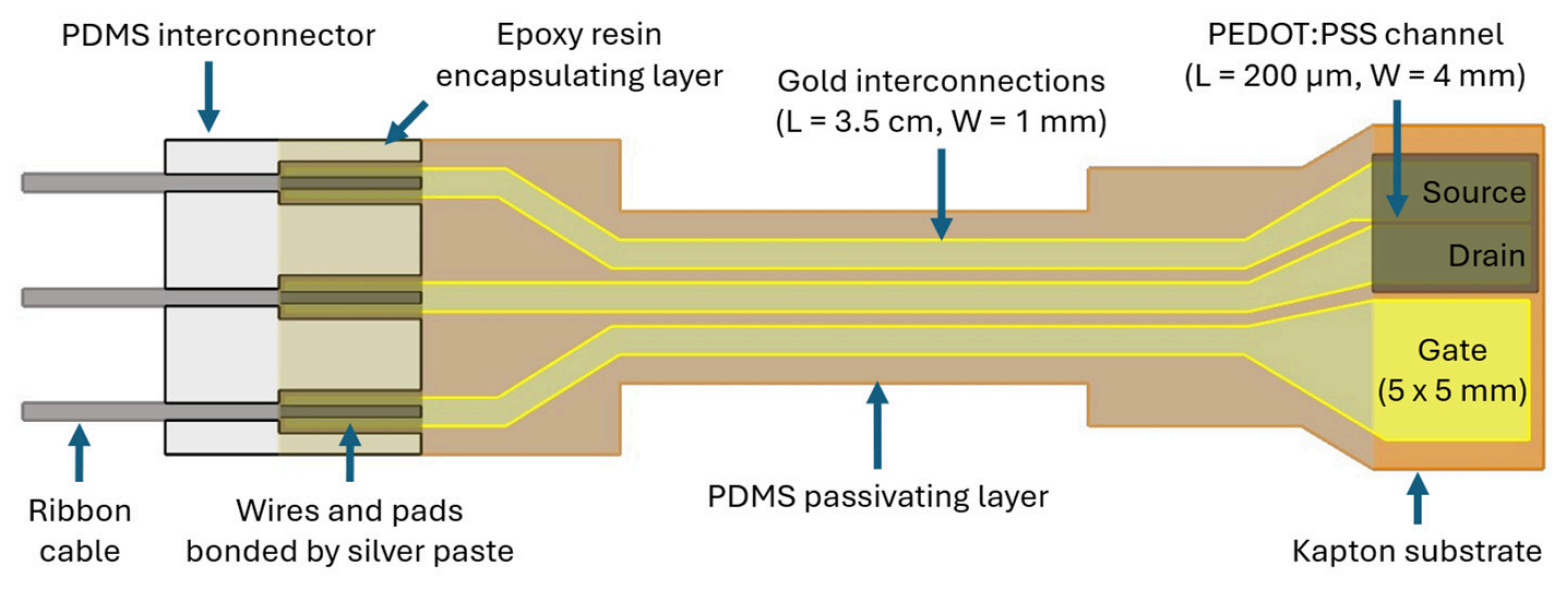
Design of the OECT setup, realized with Rhinoceros CAD software.

The OECT setup (Figure 6) was customized for integration into a 12-well culture plate and for interfacing with the breadboard. A 25 µm-thick flexible Kapton substrate (DuPont, Wilmington, DE, USA) was selected to allow sensor bending within the wells. Gold was employed for the OECT electrodes, interconnections and contact pads, whereas PEDOT:PSS was used for the channel [18]. This region was designed with a length of 200 µm and a width of 4 mm, while the gate was designed as a 5 mm × 5 mm gold electrode. A polydimethylsiloxane (PDMS) layer was used for gold passivation. All materials were chosen for their biocompatibility and reliable operation in biological environments. Following the approach of Fallager et al. [19], a guide-patterned PDMS mold was used as an interconnector between the pads and the stripped wires of a 10 cm-long ribbon cable. Silver paste was used to bond the pads and wires, which were then encapsulated with epoxy resin for protection against cell incubator humidity (95% RH). This interconnection block was conceived to prevent short circuits between the bonded contacts and to provide mechanical stability. All the aforementioned structures were designed using Rhinoceros CAD software (v8, Robert McNeel & Associates, Seattles, WA, USA). A wiring setup was engineered for signal transmission between the OECT setup and the breadboard, consisting of insulation-displacement contact (IDC) connectors linked by a 1 m-long ribbon cable (as shown in Section 3.1). Ferrite rings enclosing the cable ends were used for EMI attenuation. This setup was tailored for communication through a cell incubator and for allowing operators to disconnect the sensorized plate from the platform during biological procedures.

### 2.2. Hardware Fabrication

**Figure 7:**
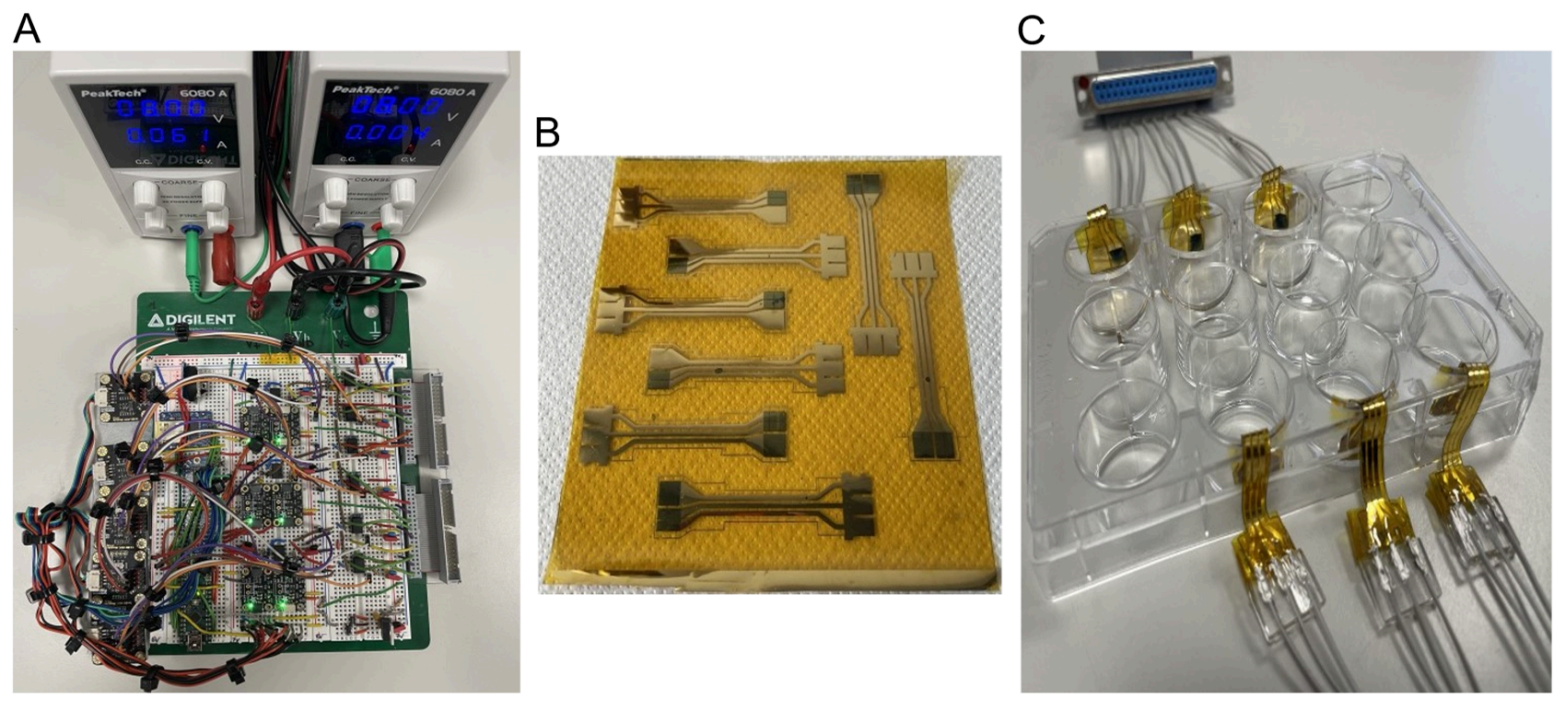
(**A**) Assembled SMU circuit on the breadboard. (**B**) Fabricated OECTs prior to geometry patterning by laser marking. (**C**) Developed OECT setup integrated into a standard culture plate.

The designed circuit was assembled on a 230 mm × 175 mm breadboard (Digilent, Pullman, WA, USA), powered by the PSU via screw terminals (Figure 7A). ADCs and connectors were integrated into the breadboard using 3D-printed holders fixed to the breadboard sides. Due to space constraints, each voltage-driving channel was wired to two DUTs, as described in Section 2.1.1. The gold paths of the OECTs were fabricated according to the following cleanroom-based process (Figure 7B). First, a 10 nm-thick Ti adhesion layer and a 100 nm-thick Au layer were sequentially deposited on Kapton by e-beam evaporation (FC-2000 evaporator, Ferrotec Temescal, Livermore, CA, USA) [18,20]. Then, the path layout was transferred to the substrate by photolithography: The AZ1518 positive photoresist (MicroChemicals GmbH, Ulm, Germany) was spin-coated (≈1.5 µm-thick at 4000 rpm, 30 s), soft-baked (110 °C, 60 s), exposed to the UV laser beam pattern at 468 mJ/cm^2^ (LW405 laser writer, Microtech, Palermo, Italy), and developed for 3 min in AZ400K developer (MicroChemicals GmbH) diluted in distilled H_2_O (1:4 ratio). Finally, the exposed Ti-Au regions were removed by wet etching using TechniEtch ACI2 etchant (MicroChemicals GmbH) for Au (4 min treatment) and a solution of NH_3_ diluted in H_2_O_2_ for Ti (1:3 ratio, 2 min treatment), respectively. The OECT channel was fabricated according to the following protocol [20]. Pristine PEDOT:PSS (Clevios PH1000, Heraeus, Hanau, Germany) was sonicated for 30 min, filtered and mixed with ethylene glycol (5% *v*/*v*) and (3-glycidoxypropyl)trimethoxysilane (GOPS) (1% *v*/*v*) to improve conductivity and stability, respectively. The obtained solution was diluted with distilled water (100% *v*/*v*) to decrease its viscosity to an appropriate range (1–10 cP) for Aerosol Jet Printing deposition (Aerosol Jet 200 Series printer, Optomec, Albuquerque, NM, USA). After treating the substrate with ozone for 10 min to enhance wettability, the OECT channel was printed by setting a fly velocity of 2 mm/s, a sheath gas pressure of 35 SCCM, a carrier gas pressure of 25 SCCM, a plate temperature of 60 °C, and an ultrasonic atomizer current of 0.5 mA, followed by annealing at 120 °C for 30 min. The passivating PDMS layer was deposited by spin-coating (≈100 µm thick at 1000 rpm, 30 s), masking the OECT sensing region and pads with dicing tape, which was subsequently stripped. The sensor layout was patterned by CO_2_ laser marking (Laser Slider, Microla Optoelectronics, Torino, Italy) with a power of 12% (6 W), a frequency of 1500 Hz and a scan velocity of 50 mm/s. Pads and ribbon cable wires were bonded by aligning them on the interconnector guides and by applying silver paste, followed by curing at 60 °C for 1 h. The bonded region was encapsulated by mixing epoxy resin with the hardener (1:1 ratio), waiting 1 h for partial curing, pouring the obtained gel over the bonded contacts and letting the gel completely cure overnight. Figure 7C shows the developed OECT setup.

### 2.3. Software Development

**Figure 8:**
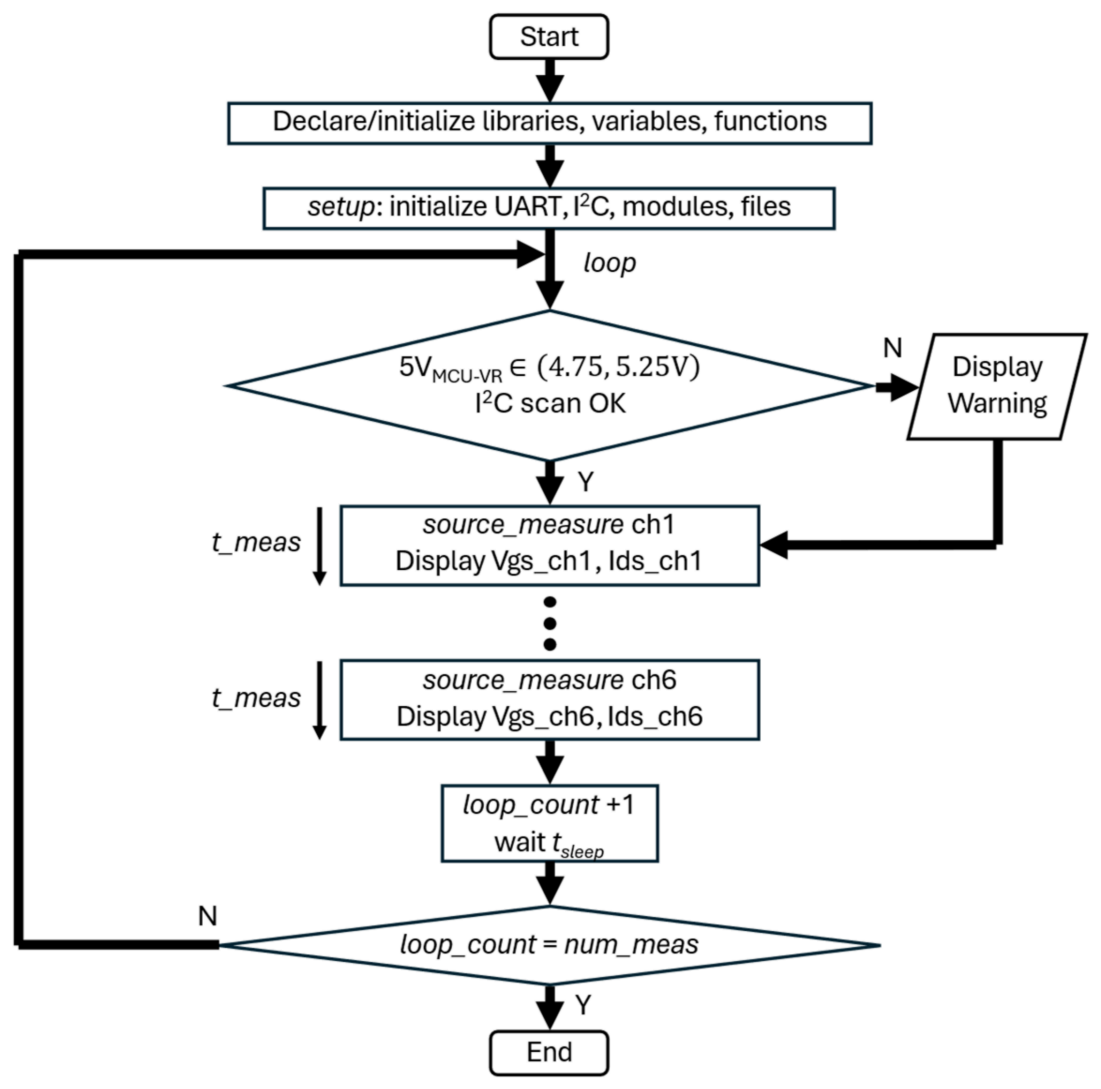
Execution flow of the developed Arduino programs.

Programs defining SMU operations (Arduino sketches) were written in the open-source Arduino Programming Language (a C/C++ dialect). The user-friendly Arduino IDE (v2.3.6) was used for library installation, sketch writing and compilation, firmware upload, and serial communication between the Arduino Nano and the PC. The execution flow of the sketches is depicted in Figure 8 and summarized as follows. It starts with the declaration and initialization of software libraries, modules, variables and functions. The Arduino setup() function initializes communication protocols, SD card data files (in CSV format), and modules. The I^2^C clock frequency was set to 400 kHz (fast mode) and the UART speed to 115,200 baud, options chosen as a compromise between data rate and communication stability. Within the Arduino loop() function, after power supply and I^2^C checks, the developed source_measure() function is executed channel by channel. For each OECT, the function applies the V_ds_ and V_gs_ signals, buffers the I_ds_, V_gs_, and timestamp values over the defined channel measurement period, logs them to the data files, and then proceeds to the next channel. Data can be accessed after the completion of measurements on all channels by transferring the SD card from the adapter module to the PC. The developed sketches, documented through in-code comments, are open-source and can be found in Supplementary Materials.

### 2.4. Keysight B2912A SMU Configuration

The proposed SMU was benchmarked against the commercial B2912A SMU (Keysight Technologies), which was presented in the Introduction. The instrument was configured with the same settings as the developed system: It was set to two-wire mode, with a cutoff frequency of 145 Hz and with the same time, voltage, and current settings as the proposed SMU at each test. The Quick IV Measurement Software (v1.0.2538.6810, Keysight Technologies) was used for B2912A configuration and measurements.

## 3. Results and Discussion

### 3.1. SMU Platform Development

**Figure 9:**
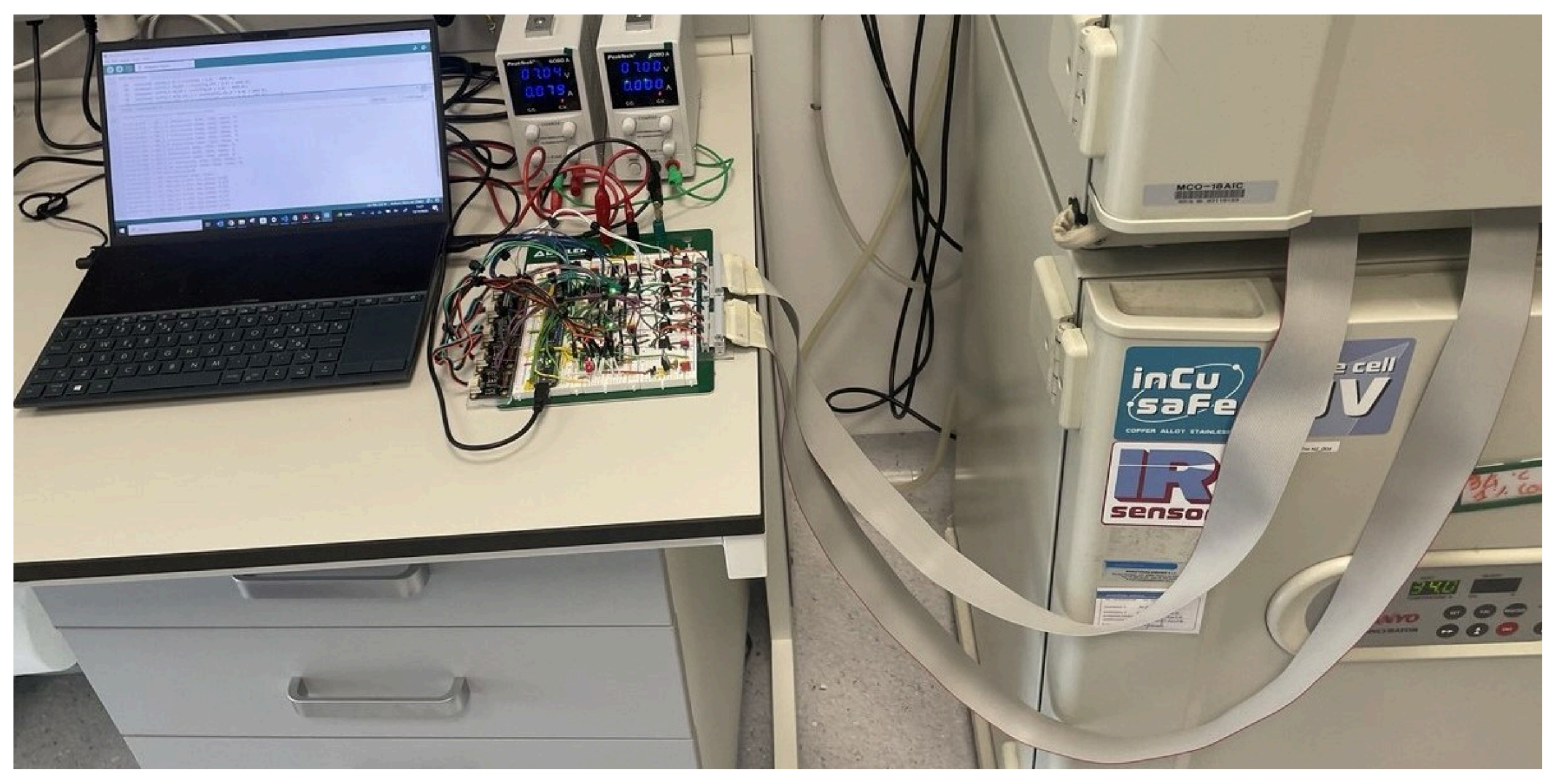
SMU platform operating in a biological laboratory. The breadboard is connected via ribbon cables to the OECT setup, which is integrated into the culture plate and operated within the cell incubator.

Figure 9 shows the developed SMU platform operating in a biological laboratory. The equipment is portable, fully integrated and optimized for in vitro operation. The system supports continuous and automated multi-OECT monitoring. Components are low-power and the analog circuitry operates at ~1 V, consuming a total average power of 1 W, which is suitable for long-term measurements. Per-channel voltage and current resolutions are 1.22 mV and 78 nA, respectively, and the sampling rate is 2.5 ms. These electrical features allow for the characterization of a wide range of OECT biosensors, such as those reported in recent reviews [21,22]. Since the maximum I_ds_ is approximately 10 mA, the 1 Ω shunt resistor ensures a maximum voltage drop of ≈10 mV across the V_ds_ signals, which negligibly perturbs the driving voltages. The SD card enables PC-independent data collection without memory constraints, thereby overcoming the limited 2 kB SRAM of the Arduino Nano. The three Arduino Nano serial communication interfaces (I^2^C, UART/USB, SPI) facilitate multi-sensor integration. The total material cost of the platform is approximately €400, yielding a cost-effective instrument. The complete bill of materials and related costs are provided in Table S1 of Supplementary Materials.

### 3.2. Calibration

By operating within the linear region of converters (20–80% of FSR), the output–input relationship of the driving and sensing subsystems can be modeled with a linear transfer function: Output = Gain ∙ Input + Offset, where Gain (ideally 1) and Offset (ideally 0) are the slope and intercept, respectively. Due to tolerances, Gain and Offset errors arise and can be compensated by SW calibration [23]. The subsystems were calibrated based on converters’ linear regions and OECT working ranges. For example, for source-voltage drivers, offsets were measured across a 47 Ω test resistor (R_t_) to simulate the OECT channel resistance. The zero-scale (ZS) and full-scale (FS) voltages (V_ZS,ideal_ = 1 V, V_FS,ideal_ = 4 V) were measured with R_t_ = 100 Ω and 470 Ω, respectively, to simulate I_ds_ ≈ 10 mA [20]. The 1 mV-resolution Fluke 113 digital multimeter (Fluke, Everett, WA, USA) was used for voltage acquisition, and the 10 mV-resolution PeakTech 6080A tunable power supply (PeakTech) was used for voltage application. Output data are reported in Table 3 and Table 4. Overall, Offset and Gain errors are within 1 mV and 1% for the voltage subsystem and within 0.2 mA and 5% for the current subsystem, respectively. These errors can be mainly attributed to the contribution of R_s_ and the tolerance of G_INA_, as well as voltage reference variations [23].

### 3.3. Hardware Characterization

#### 3.3.1. Residual Offset and Noise

**Figure 10:**
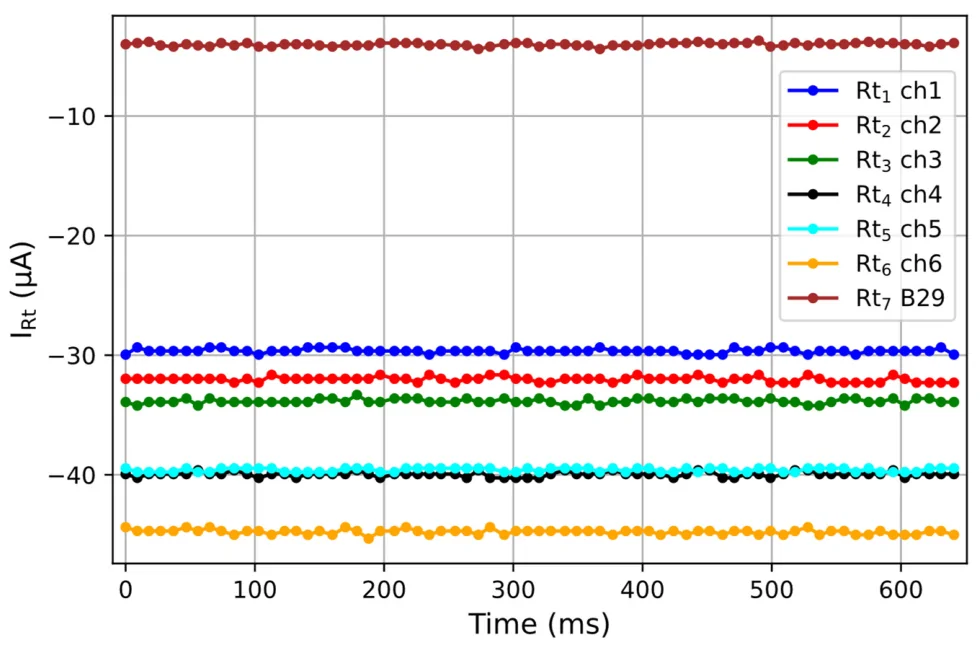
Residual offset and noise for each channel of the prototype and for B2912A, measured with test resistors R_t_ of 47 Ω. FSR = 20.48 mA, V_Rt_ = 0 V, SPS = 128, acquired samples = 70.

After calibration, residual offset and noise were assessed with R_t_ = 47 Ω (OECT channel resistance simulation), FSR = 20.48 mA (OECT I_ds_ range), null input voltages (V_Rt_ = 0 V), SPS = 128 (default option), and acquired samples = 70. The offset was calculated as the average of the acquired I_Rt_ current samples, computed according to Equation (1). The noise was defined and calculated as the root mean square error (RMSE) of the samples with respect to the offset. As reported in Figure 10 and Table S2, residual offsets between −30 and −45 µA and noise values between 155 and 210 nA were measured despite calibration. They can be attributed to parasitic contact resistances [7], ground loops of the breadboard [24], test resistor tolerances and temperature variations of components [15]. Even the commercial B2912A was affected by an offset of 4 µA and a noise level of 134 nA, with the latter parameter being slightly lower than that of the prototype. To compensate for inter-channel and time-dependent offset variability, a dynamic calibration method was implemented. In post-processing, for each characterization cycle and channel, raw current data are corrected by subtracting the corresponding offsets acquired prior to signal application. This guarantees inter-channel data comparability and independence from breadboard-related offsets. The method is visually represented in Figure S1. However, the average offset of 32 µA establishes a lower limit for the I_ds_ operating range, which is nevertheless compatible with most OECT biosensors, as previously reported [21,22].

#### 3.3.2. Test Resistor Characterization

**Figure 11:**
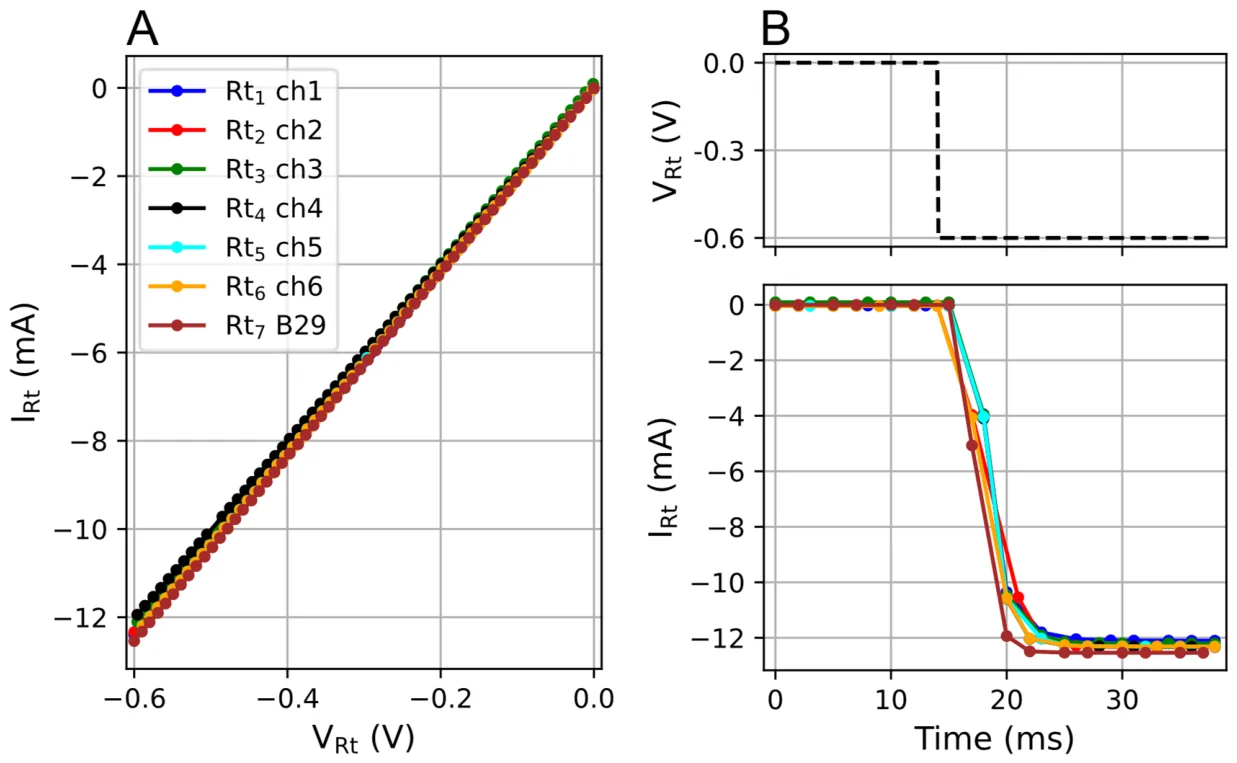
Test resistor characterization (R_t_ = 47 Ω) for each channel of the prototype and for B2912A. (**A**) I–V characterization. V_Rt,sweep_ = 0–−0.6 V, FSR = 20.48 mA, SPS = 128, settle time = 200 ms, step size = 10 mV. (**B**) Time response characterization. V_Rt,step_ = 0–−0.6 V, FSR = 20.48 mA, SPS = 860.

The system was first tested with the previously utilized 47 Ω resistor, which has a well-known behavior. Test settings were based on OECT working ranges and were identically applied to each channel and to B2912A. Currents were computed according to Equation (1). The I–V characterization was executed by sweeping V_Rt_ between 0 and −0.6 V (OECT V_ds_ range [20]) and by applying FSR = 20.48 mA, SPS = 128, settle time = 200 ms (sufficient for OECT stabilization) and step size = 10 mV (a compromise between resolution and slope fluctuations [13]). The acquired I_Rt_–V_Rt_ channel curves, visualized in Figure 11A, agree with the expected trend represented by the B2912A linear characteristic. A quantitative comparison of the curves with respect to the B2912A reference is summarized in Table 5. For each channel, the table reports the mean absolute percentage error (MAPE), the root mean square error normalized to the FSR (NRMSE_FSR_), the coefficient of determination (R^2^), and the gain (conductance) relative error (G ε_r_). The average MAPE and NRMSE_FSR_ across channels are 4.2% and 1.3%, respectively, with a maximum MAPE of 8.1%. All R^2^ values reach 0.999, confirming the linearity and agreement of the curves. The average gain error is 1.8%, with a peak deviation of 4.1%. Overall, these metrics demonstrate the hardware’s functional consistency and reasonable accuracy for I–V characterization. Gain discrepancies are attributable to breadboard parasitic effects, temperature variations and component tolerances, as previously outlined. The obtained gain errors establish the current sensitivity limit of the instrument.

For the time-response characterization, V_Rt_ was stepped from 0 to −0.6 V with SPS = 860 (highest configurable value), resulting in a sampling rate of ≈2.5 ms, which was likewise set on the B2912A. The obtained responses, plotted in Figure 11B, are consistent and follow the expected behavior: currents are ≈0 mA at V_Rt_ = 0 V and stabilize at ≈−12.3 mA after a steep transient. Steady-state channel currents differ by approximately 200 µA from those of B2912A. This variation can be attributed to LM358, which simultaneously drives two loads while having a maximum output current of 20–30 mA, as specified in the component datasheet. Rise times (t_r_) were calculated as the time required for the current to rise from 0% (last sample before the step) to 100% (first stable sample). As shown in Table 5, they are equal (t_r_ ε_r_ = 0%) to or higher by 1–2 ms (t_r_ ε_r_ = 10–20%) than those obtained with the B2912A (10 ms). These delays are presumed to arise from operating the ADS1115 in single-shot mode, which enters a low-power state between acquisitions but requires per-sample conversion, as noted in the component datasheet.

#### 3.3.3. Long-Term Continuous Acquisition

**Figure 12:**
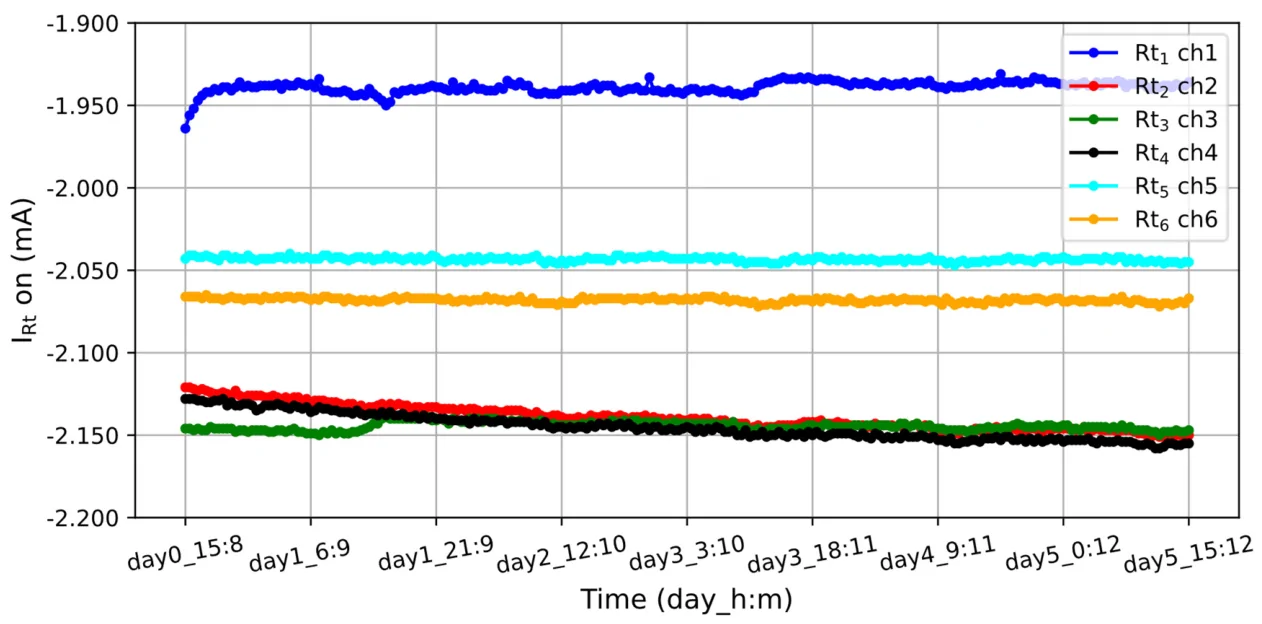
Continuous time response characterization of test resistors (R_t_ = 47 Ω). The I_Rt,on_ values result from the application of V_Rt,step_ = −0.1 V in a 5 day acquisition with 30 min sampling intervals. The presented data were obtained through dynamic offset calibration applied in post-processing. FSR = 2.56 mA, SPS = 860.

The continuous recording capability and long-term stability of the prototype were evaluated by running an automated time response characterization every 30 min over 5 days. These temporal parameters were selected to replicate the timelines of in vitro experiments. Configurations were the same as those for the time response characterization of the test resistors (Section 3.3.2), adjusting V_Rt,step_ = −0.1 V and FSR = ±2.56 mA (lowest FSR option) to simulate standard V_ds_ and I_ds_ ranges in continuous OECT-based cell monitoring [25]. The dynamic offset calibration (Section 3.3.1) was applied to the raw I_Rt_ data, as exemplified in Figure S1. The resulting I_Rt,on_ values (i.e., responses to V_Rt,step_) acquired for each channel at 30 min sampling intervals are plotted in Figure 12. These records demonstrate the system’s capability to execute continuous characterization over durations comparable to in vitro experiments. Current curves display an overall steady-state behavior, reflecting the temporal stability of the instrument. To quantify this stability and evaluate current fluctuations, Table 6 reports the average values, temporal drift and coefficient of variation (CV) of the I_Rt,on_ data. The average drift and CV across all channels are 0.7% and 0.2%, respectively, confirming the long-term stability of the system and negligible current fluctuations. Inter-channel discrepancies in average currents are evident, although they remain within 200 µA. They can be attributed to channel gain errors (as discussed in the previous section) and to the 5% tolerance of the utilized 47 Ω CFR25 test resistors.

### 3.4. OECT Characterization

**Figure 13:**
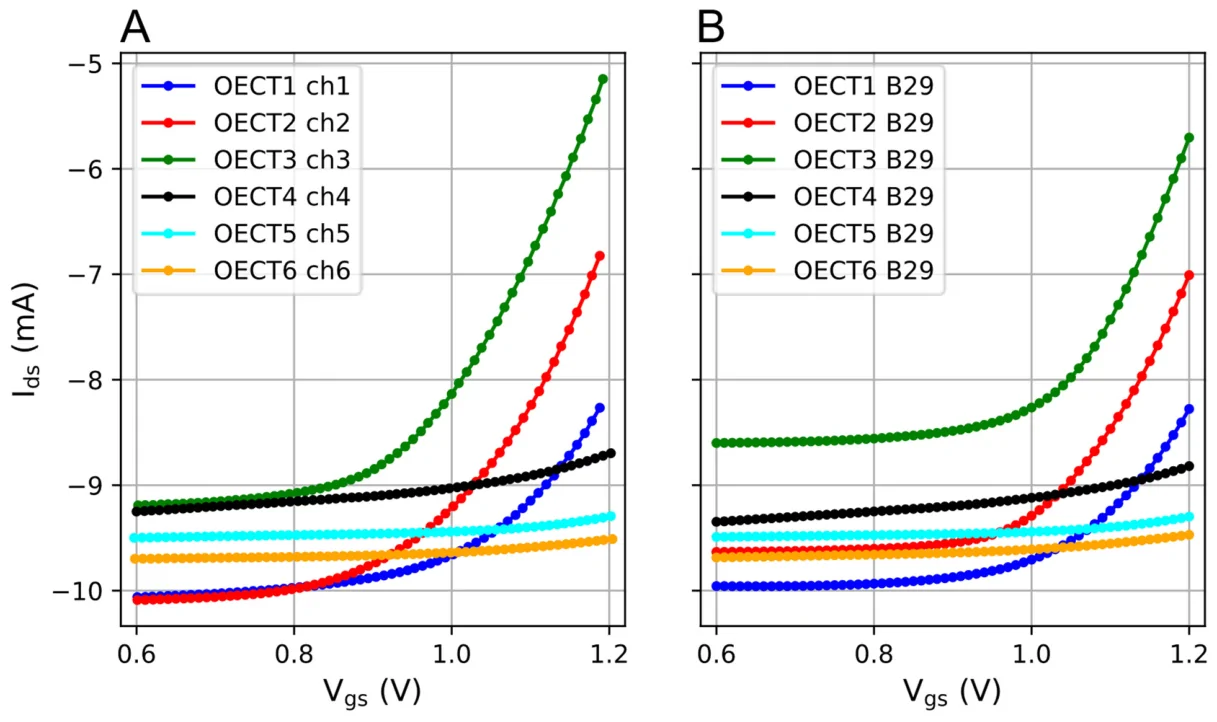
I_ds_–V_gs_ characterization of the six-OECT setup executed with the developed SMU platform (**A**) and with B2912A (**B**). V_gs,sweep_ = 0.6–1.2 V, V_ds_ = −0.3 V, FSR = 20.48 mA, SPS = 128, settle time = 200 ms, step size = 10 mV.

**Figure 14:**
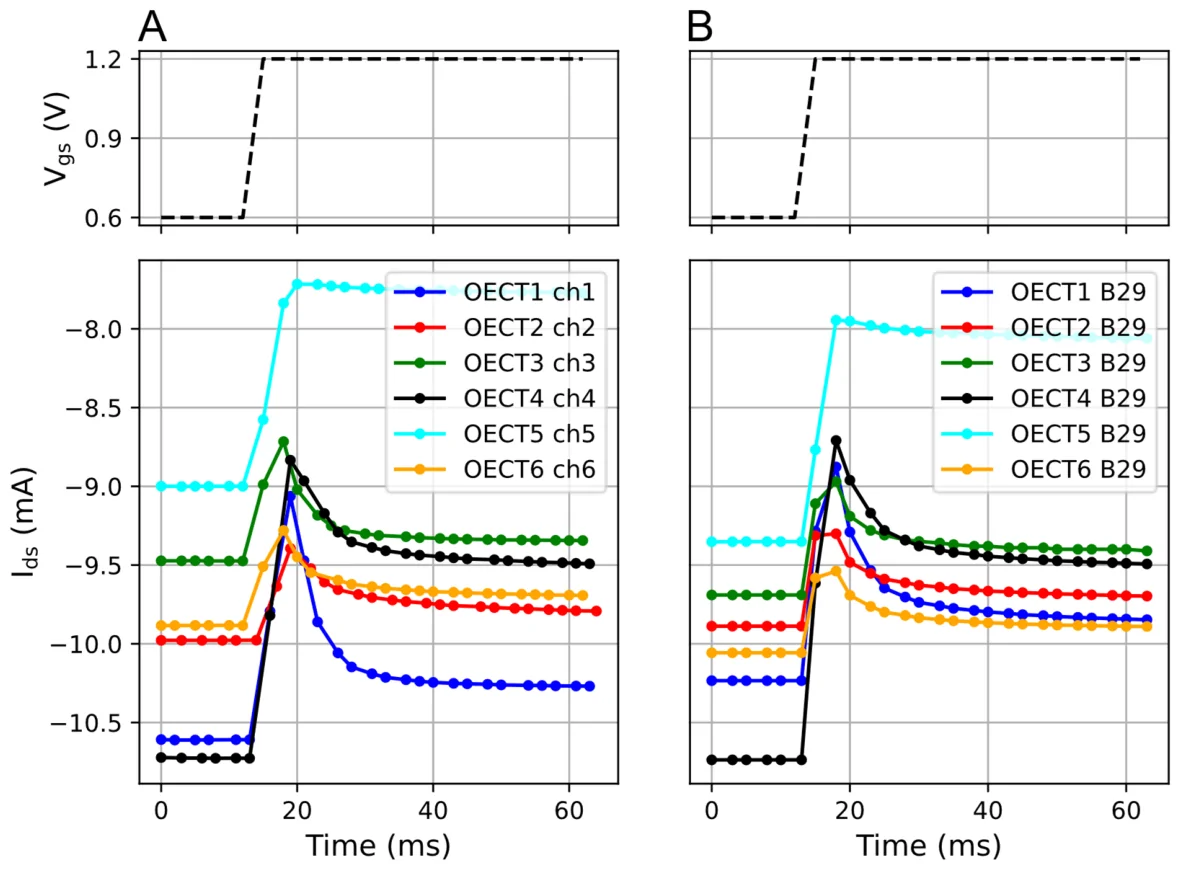
Time response characterization of the six-OECT setup executed with the developed SMU platform (**A**) and with the B2912A SMU (**B**). V_gs,step_ = 0.6–1.2 V, V_ds_ = −0.3 V, FSR = 20.48 mA, SPS = 860 (sampling rate = 2.5 ms).

The system’s OECT characterization performance was validated by comparing the acquired transfer characteristics (I_ds_–V_gs,sweep_) and time responses (I_ds_(t)–V_gs,step_) with those obtained with B2912A. Experimental conditions were the same as those for the test resistor characterization (Section 3.3.2), with the introduction of OECT-related settings: V_ds_ was fixed at −0.3 V and V_gs_ was swept between 0.6 and 1.2 V for the transfer characteristic and stepped from 0 to 1.2 V for the time response. These higher-value voltages were applied to better observe current variations in the fabricated OECTs. I_ds_ currents were computed according to Equation (1), and their offset was corrected as described in Section 3.3.1. The OECT setup (Figure 7C, Section 2.2) was tested in an electronics laboratory by placing 2 mL of 1X phosphate-buffered saline (PBS) solution into each well (as done in [26]). In the prototype measurements, the OECT setup was wired to the breadboard as shown in Figure 9 (Section 3.1) and characterized as explained in Section 2.3. For B2912A, the SMU cables were manually wired and switched to the connector pins of each tested OECT for each characterization. Results are reported in Figure 13 and Figure 14. First, Figure 13A and Figure 14A demonstrate that the developed SMU is able to consistently perform sequential transfer characteristic and time response characterizations on OECTs. Corresponding curves are qualitatively consistent between the two instruments, allowing the different trends to be clearly distinguished.

Table 7 reports statistical parameters for the quantitative comparison between the I_ds_–V_gs_ channel curves and the B2912A reference curve. The average MAPE and NRMSE_FSR_ are 2.4% and 1.1%, respectively, in alignment with the values obtained for the test resistors (Section 3.3.2). The average R^2^ of 0.983 reflects the visual deviations observed between corresponding curves. Relative errors of typical OECT metrics are also reported for comparison, namely the peak transconductance error (g_m,max_ ε_r_) for the transfer characteristic and the rise time error (t_r_ ε_r_) for the time response curves. The average and maximum g_m,max_ ε_r_ reach 19% and 36%, whereas those for t_r_ ε_r_ are 12% and 33%, respectively. These errors exhibit higher values and channel-to-channel fluctuations compared to those in the test resistor analysis. Unlike commercial resistors, the fabricated OECTs contribute to the observed discrepancies. As reported in Figure S2 and Table S3, B2912A characterizations conducted on three fabricated OECTs across two measurement rounds exhibit deviations comparable to those obtained between the two instruments. Indeed, repeated measurements impact OECT stability through ion channel doping and electrical stress [26]. Moreover, ambient temperature fluctuations alter PBS samples and OECT conditions, affecting sensor behaviour.

### 3.5. OECT Long-Term Continuous Monitoring

**Figure 15:**
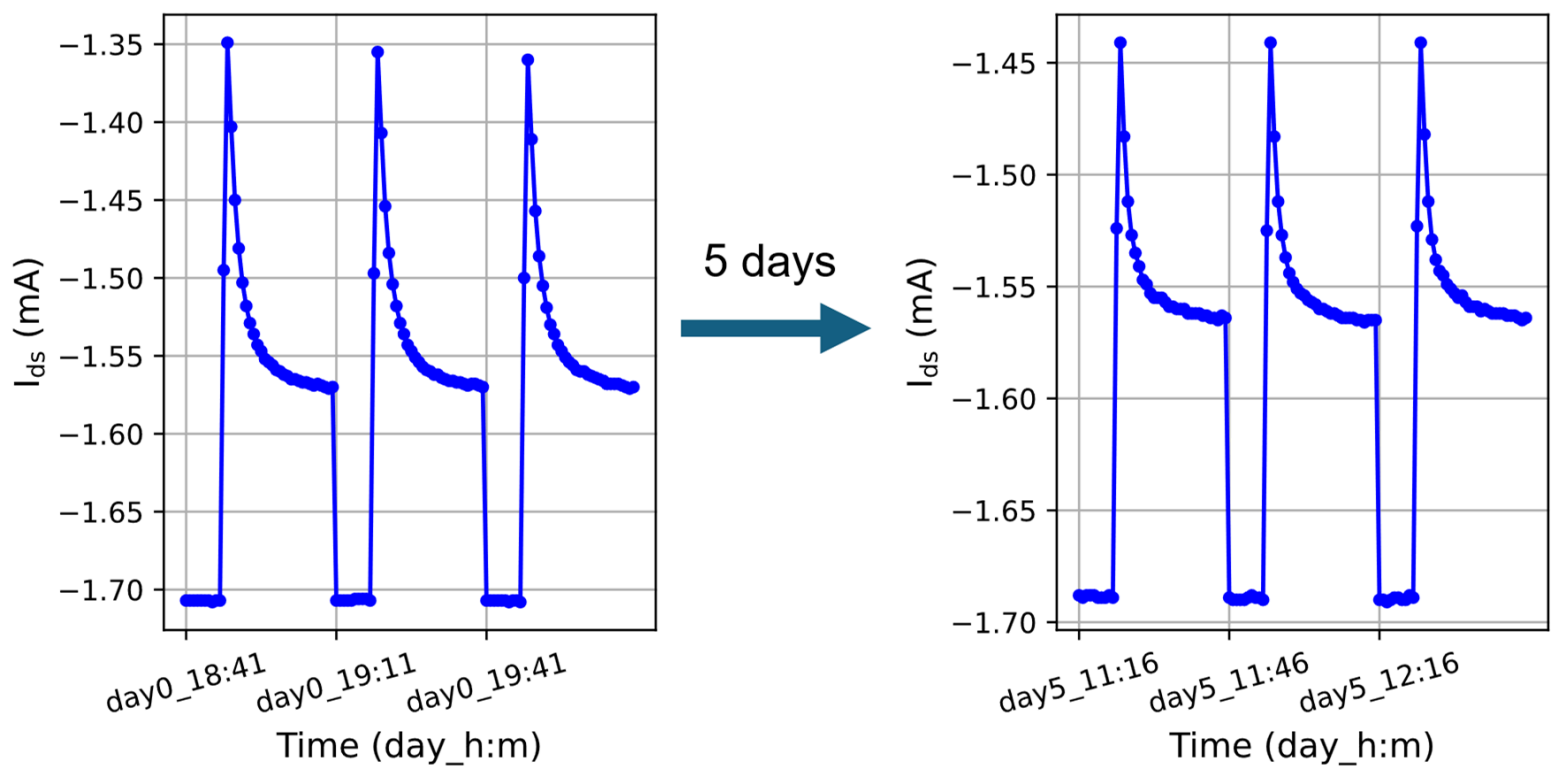
First and last recorded time responses for one representative OECT of the setup in a 5 day continuous experiment with 30 min sampling intervals. V_gs,step_ = 0–0.3 V, FSR = 20.48 mA, SPS = 860 (sampling rate = 2.5 ms), sampling interval = 30 min, experimental period = 5 days.

**Figure 16:**
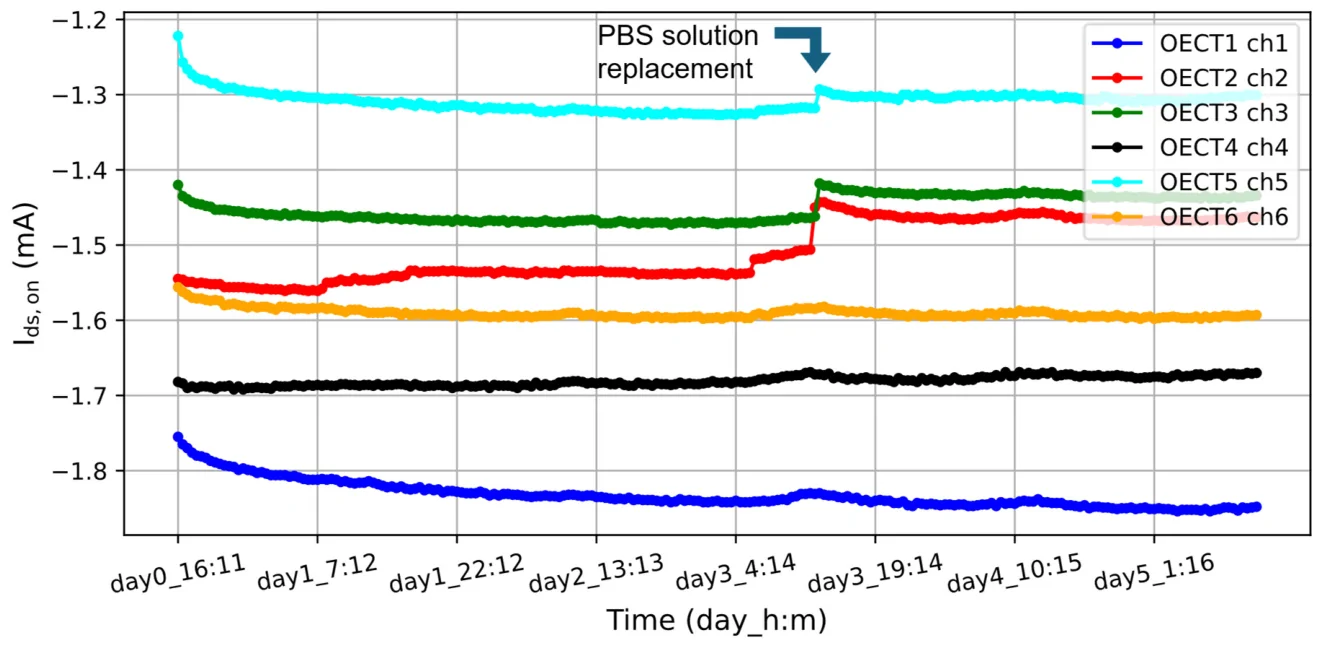
Continuous time response characterization of OECTs. The I_ds,on_ values result from the application of V_ds_ = 0.1 V prior to V_gs,step_ = 0.3 V in a 5 day acquisition with 30 min sampling intervals. The presented data were obtained through dynamic offset calibration applied in post-processing. FSR = 2.56 mA, SPS = 860 (sampling rate = 2.5 ms). To simulate the culture medium change, the PBS solution was replaced on the third day.

The primary aim of this work was to develop an operator-independent SMU for long-term and continuous monitoring of OECT biosensors. For this validation, the platform was set as described in the previous section and operated as in Section 3.3.3, with the introduction of V_gs,step_ = 0.3 V [27]. Figure 15 shows the first three and last three acquired time responses of a representative OECT in the setup. Figure 16 displays the I_ds,on_ curves recorded every 30 min (i.e., I_ds_ values at V_ds_ prior to V_gs,step_ application). These data confirm the instrument’s ability to execute automated, continuous and long-term multi-OECT characterizations. Thus, the platform can be used to monitor the evolution of in vitro models through time-varying OECT metrics, such as the time constant (τ) of the I_ds_ transient. Overall, the I_ds,on_ data exhibit a stable trend, consistent with the absence of stimuli applied to the PBS samples. To simulate the standard in vitro procedure of culture medium change, the 2 mL PBS sample in each well was replaced on the third day. Clearly, the new samples strongly affected the I_ds,on_ values of OECTs 2, 3, and 5, while minimally influencing OECTs 1, 4, and 6. This effect can be attributed to the varying stability among the PEDOT:PSS films, which responded distinctly upon interfacing with the fresh solution. This effect must be taken into account in continuous in vitro experiments for proper data interpretation.

As reported in Table 8, the average temporal drift and CV of the I_ds,on_ data across all channels are 3.5% (with a maximum of 6.5%) and 1.1%, respectively. The higher current drift and fluctuations compared to those in the test resistor analysis are expected due to OECT stability degradation caused by bias stress, as addressed at the end of the previous section.

## 4. Conclusions

In this work, we have developed a low-cost SMU platform for long-term and continuous characterization of OECT biosensors, combining portability, customization for the in vitro environment, multi-sample monitoring and operator independence. The system integrates open-source, fully documented and user-friendly hardware and software components, supporting prototyping and customization. The aim of the present work was not to develop an instrument with superior electrical performances compared to commercial or advanced low-cost SMUs. Rather, its main contribution is the integration of multiple features into a single piece of equipment, specifically dedicated to in vitro OECTs. Unlike previous works and standard SMUs, the platform features a six-channel acquisition architecture, continuous and automated I–V and time response measurements with embedded data storage (eliminating the need for additional hardware or user intervention), and long-term acquisition capability, addressing the multi-day duration of biological experiments. Focusing on in vitro compatibility, the instrument is equipped with a tailored setup for laboratory procedures and for OECT integration into standard culture plates.

In terms of electrical specifications, the system exhibits an operating range of 5 V/12 mA, a resolution of 1.22 mV/78 na, a sampling rate of 2.5 ms and an average power consumption of 1 W. These electrical features enable the application of the platform to other classes of organic transistor biosensors, such as Electrolyte-Gated Organic Field-Effect Transistors (EGOFETs) and Organic Charge-Modulated Field-Effect Transistors (OCMFETs). Their low operating voltages (<2 V) and similarity in I–V behaviour to OECTs enable their characterization by the developed SMU. In contrast, the system is incompatible with Organic Field-Effect Transistors (OFETs) as they typically operate at higher voltages (~10 V) and frequencies (~MHz) [1,2,4].

We have evaluated the characterization performance of the system through I–V and time response measurements on commercial resistors and on a six-OECT setup interfaced with PBS, comparing the outcomes with those obtained by the Keysight B2912A SMU. The obtained characterization curves and quantitative metrics demonstrate correct functionality and consistency with B2912A. The average noise, offset, and gain errors are approximately 180 nA, 40 µA, and 2% (at FSR = 20.48 mA), respectively. These values establish the minimum measurable current and sensitivity limit for OECTs. The continuous and long-term acquisition performance of the platform was validated on commercial resistors and OECTs through a 5 day test with a 30 min sampling interval. Data demonstrate the continuous recording capability of the platform and its stability over biological experiment durations, yielding reasonable current drift and CV. Therefore, the achieved results suggest that for in vitro continuous characterization of OECT biosensors, the proposed solution is a cost-effective option compared to expensive, low channel count and bulky conventional SMUs.

We are aware of the electrical constraints of the instrument, and ongoing work is focused on its improvement. To attenuate offset, gain and noise inaccuracies and prevent contact degradation, the breadboard will be transitioned to a PCB. The octal-channel DAC7578 module (Texas Instruments, Adafruit Industries) will replace the single-channel MCP4725 DACs to save space and reduce the number of required I^2^C channels. To expand the measurement capabilities towards faster and lower-current OECT biosensors, the 860 SPS ADS1115 will be replaced by the 3300 SPS ADS1015 module (Texas Instruments, Adafruit Industries), achieving a sampling rate of less than 1 ms. Despite adopting a lower-resolution ADC, the I_ds_ resolution, which is proportional to G_INA_ and R_s_ (Equation (1)), can be maximized to 12.5 nA by increasing R_s_ to 10 Ω and G_INA_ to 1000 (by means of R_G_, Equation (2)). However, with these settings, the I_ds_ range would be limited to 100 µA to remain within the maximum FSR of ADS1015. To address this trade-off, following the approach of Galanti et al. [9], sockets for interchangeable R_G_ and R_s_ resistors can be implemented to adjust current ranges and resolutions according to the tested OECT. Finally, we are developing a protective PCB enclosure against environmental agents and a customized power supply to comply with biological laboratory regulations. Still, first-quadrant and voltage-controlled-only operation remain the primary hardware constraints, requiring major architectural changes.

As this work evaluated instrument performance with PBS and its integration within a biological environment, a comprehensive validation by means of standard in vitro experiments is required. In this context, instrument usability will be assessed by end-users in terms of measurement execution, data access, OECT setup integration into culture plates and handling during in vitro procedures. OECTs will be tested for biocompatibility and suitability with standard sterilization methods. Following these preliminary validations, the platform will be employed in continuous live-cell monitoring experiments to confirm its electrical and long-term performance for cell-based applications. In this context, the platform aims to target use cases such as OECT-based monitoring of dynamic tissue responses under external stimuli [27,28]. Furthermore, given the emerging role of OECTs in point-of-care testing (POCT) [29], future engineering iterations could address the present lack of automation of sample treatment, sensing and data analysis. Drawing inspiration from the fully automated POCT architecture developed by Zhou and colleagues [30], the platform could move towards POCT applications by incorporating two dedicated subsystems for automated sample processing and embedded data analysis.

## Figures and Tables

**Table 1:** Key features of the present SMU platform and reported prototypes.

Ref.	Application	Channels	Measurements	External HW	Open-Source SW	Cost (€) *
Present SMU	OECT biosensors	6	I–V, time response, continuous, long-term	Power supply	Yes	290
[5]	LEDs	1	I–V	PC, DUT setup	No	70 **
[7]	Commercial electronic devices	1	I–V	Power supply, PC	No	58
[8]	Photovoltaic devices	1	Periodic I–V	Power supply, DUT setup	Yes	69
[9]	Graphene FETs	2	I–V	Power supply, CPU, PC, DUT probe station	No	345
[10]	Ionic liquid-gated FETs	1	I–V	Power supply, DUT probe station	No	100 **
[12]	Photodiodes and phototransistors	1	I–V	PC, DUT setup	Yes	30 **
[13]	Commercial electronic devices	1	I–V, AC time response	PC, DUT setup	No	80 **
[14]	Resistive switching devices	1	Pulsed I–V, time response, HSL	Power supply, DAQ, DUT probe station	No	50 **
[15]	FET biosensors	3	I–V	PC, DUT setup	No	197

* Excluded external HW. ** Not directly reported; calculated from the provided information.

**Table 2:** Main electrical specifications of the present SMU platform and reported prototypes.

Ref.	V Range (V)	V Resolution (mV)	I Range (mA)	I Resolution (nA)
Present SMU	5	1.22	12	78
[5]	5	1.22	150 *	N/A
[7]	20	0.76	2 × 10^3^	1 × 10^6^
[8]	12	1.22	70	460 × 10^3^
[9]	10	0.001	0.1	0.490
[10]	5	1.22	12	1
[12]	5	100 *	0.025 *	24 *
[13]	5	1.22	15	8 × 10^3^
[14]	10	0.03 *	25	1 *
[15]	20	100	1 × 10^3^	10 × 10^6^

* Not directly reported; calculated from the provided information.

**Table 3:** Offset and Gain errors for each subcircuit of the voltage driving and sensing subsystems. Test conditions are based on converters’ linear regions and OECT voltage ranges.

Voltage Driving/Sensing	Offset (mV)	Gain Error (%)	Conditions
Driving Vs_1	0.0	1.2	Offs: R_t_ = 47 Ω ZS: V_ZS_ = 1 V, R_t_ = 100 Ω FS: V_FS_ = 4 V, R_t_ = 470 Ω
Driving Vs_2	0.0	1.1	
Driving Vs_3	0.0	0.3	
Driving Vg_1	0.0	0.3	Open-circuit ZS: V_ZS_ = 1 V FS: V_FS_ = 4 V
Driving Vg_2	0.0	0.3	
Driving Vg_3	0.0	0.4	
Sensing Vgs_1	0.0	0.1	FSR = 6.144 V Open-circuit ZS: VZS = −4 V FS: VFS = 4 V
Sensing Vgs_2	0.0	0.1	
Sensing Vgs_3	0.0	0.1	
Sensing Power	−1.0	0.1	

**Table 4:** Offset and Gain errors for each channel of the current-sensing subsystem. Test conditions are based on converters’ linear regions and OECT current ranges.

Current Sensing	Offset (mA)	Gain Error (%)	Conditions
Sensing I_ch1	0.005	4.3	FSR = 20.48 mA R_t_ = 47 Ω ZS: I_ZS_ 1.85 mA FS: I_FS_ 12.5 mA
Sensing I_ch2	−0.190	3.7	
Sensing I_ch3	0.005	4.3	
Sensing I_ch4	−0.019	1.2	
Sensing I_ch5	0.000	2.1	
Sensing I_ch6	−0.130	2.2	

**Table 5:** Quantitative comparison of characterization curves measured on test resistors by the prototype with respect to the B2912A reference, as illustrated in Figure 11.

Channel	MAPE (%)	NRMSE_FSR_ (%)	R^2^	G ε_r_ (%)	t_r_ ε_r_ (%)
Ch 1	2.4	1.0	0.999	1.2	10
Ch 2	2.8	1.2	0.999	1.8	20
Ch 3	8.1	1.8	0.999	1.8	0
Ch 4	5.8	2.1	0.999	4.1	0
Ch 5	3.2	1.1	0.999	1.0	0
Ch 6	2.8	0.9	0.999	1.0	10

MAPE: mean absolute percentage error; NRMSE_FSR_: root mean square normalized to the FSR (20.48 mA); R^2^: coefficient of determination; G ε_r_: gain error; t_r_ ε_r_: rise time error.

**Table 6:** Average temporal drift and coefficient of variation (CV) of I_Rt,on_ currents acquired during a continuous 5 day time response characterization of the test resistors with 30 min sampling intervals, as illustrated in Figure 12.

Channel	Avg (mA)	Drift (%)	CV (%)
Ch 1	−1.94	1.43	0.19
Ch 2	−2.14	1.37	0.35
Ch 3	−2.14	0.05	0.12
Ch 4	−2.15	1.27	0.36
Ch 5	−2.04	0.10	0.06
Ch 6	−2.07	0.05	0.06

**Table 7:** Quantitative comparison of characterization curves measured on the OECT setup by the prototype with respect to the B2912A reference, as illustrated in Figure 13 and Figure 14.

Channel	MAPE (%)	NRMSE_FSR_ (%)	R^2^	g_m,max_ ε_r_ (%)	t_r_ ε_r_ (%)
Ch 1	0.7	0.4	0.995	8	3
Ch 2	3.1	1.5	0.976	17	33
Ch 3	5.3	2.4	0.953	6	11
Ch 4	1.4	0.6	0.999	14	13
Ch 5	2.2	1.0	0.990	36	3
Ch 6	1.7	0.8	0.987	33	7

**Table 8:** Average temporal drift and CV of I_ds,on_ currents acquired during the continuous 5 day time response characterization of the OECT setup with 30 min sampling intervals, as illustrated in Figure 16.

Channel	Avg (mA)	Drift (%)	CV (%)
Ch 1	−1.8	5.3	1.0
Ch 2	−1.5	5.3	2.6
Ch 3	−1.4	1.0	1.2
Ch 4	−1.7	0.7	0.4
Ch 5	−1.3	6.5	1.0
Ch 6	−1.6	2.4	0.4

## Data Availability

The original contributions presented in this study are included in the article and Supplementary Materials. Further inquiries can be directed to the corresponding author.

## Supplementary Materials

The following supporting information can be downloaded at: https://www.mdpi.com/article/10.3390/s26206526/s1, Table S1: Bill of materials and costs of the SMU platform; Table S2: Residual offset and noise for each channel of the prototype and for B2912A, measured with R_t_ = 47 Ω, FSR = 20.48 mA, V_Rt_ = 0 V, SPS = 128, acquired samples = 70; Table S3: Quantitative comparison of characterization curves of three OECTs obtained by B2912A in two different rounds, as illustrated in Figure S2; Figure S1: Illustration of the dynamic calibration method for correction of inter-channel, time-dependent offset. In post-processing, for each channel and characterization cycle (“c_x_” in the figure), raw currents are corrected by subtracting the corresponding offset value acquired prior to signal application, obtaining I_Rt_, _on_(c_x_) = I_Rt_, _on raw_(c_x_) − offset(c_x_); Figure S2: Characterization of three fabricated OECTs by B2912A in two different rounds. (A) I_ds_–V_gs_ characterization. V_gs_ = 0.6–1.2 V (sweep), FSR = 20.48 mA, SPS = 128, settle time = 200 ms, step size = 10 mV. (B) Time response characterization. V_gs_ = 0.6–1.2 V (step), FSR = 20.48 mA, SPS = 860 (sampling = 2.5 ms).
