# Supplementary material for: Multichannel, Portable and Low-Cost Source Measure Unit Platform for the Continuous Characterization of Organic Electrochemical Transistor Biosensors
Source: Sensors (Basel). 2026 Oct 15;26(20):6526. doi: 10.3390/s26206526 (PMC13708955; doi:10.3390/s26206526)
Supplement: Supplementary file 1 [file sensors-26-06526-s001.zip › Campanini_SMU platform_SM.pdf]

Supplementary Materials

Multichannel, portable and low-cost Source and Measure Unit platform for the continuous characterization of Organic Electrochemical Transistor biosensors.

Michele Campanini [1,2], Lorenzo Vigna* [1,2], Davide Vurro [3], Pasquale D'Angelo [3], Martina Cicolini [1,2], Simone Luigi Marasso [1,2,3], Matteo Cocuzza ,[1,2] and Alberto Ballesio [1,2,3]

1 Department of Applied Science and Technology (DISAT), Politecnico di Torino, Corso Duca Degli Abruzzi 24, 10129 Turin, Italy
2. PiQuET LAB, Piemonte Quantum Enabling Technology, Str. delle Cacce, 91, 10135 Turin, Italy
3 Institute of Materials for Electronics and Magnetism, IMEM-CNR, Parco Area delle Scienze 37/A, 43124 Parma, Italy
* E-mail: lorenzo.vigna@polito.it;

Table S1. Bill of materials and costs of the SMU platfom.

Component	Q.ty	Approx. tot. cost (€)
PeakTech 6080A PSU	2	140
ADS1115 ADC module	5	75
Breadboard	1	50
MCP4725 DAC module	6	30
Arduino Nano MCU	1	26
DS3231 RTC module	1	18
INA121 INA	6	18
TCA9548A MUX module	2	14
Ferrite ring	4	12
Ribbon cable	3 m	10
USD500S SD	1	9
MicroSD adapter	1	8
Jumper wire	100	5
Banana cable	4	5
D-sub IDC male connector	2	4
LM358 VF	3	1
IDC male connector	2	1
IDC female connector	4	1
LM317 VR	1	0.5
LED	1	0.1
R 1 Ω	6	0.1
R 511 Ω	6	0.1
C 10 μF	3	0.1
C 1 μF	13	0.1
C 100 nF	15	0.1
14001G diode	2	0.1
R 220 Ω	1	0.01
R 1 KΩ	12	0.01
R 330 Ω	2	0.01
R 220 Ω	1	0.01
**Total cost**		**428**

Table S2. Residual offset and noise for each channel of the prototype and for B2912A, measured with Rt = 47 Ω, FSR = 20.48 mA, VRt = 0 V, SPS = 128, acquired samples = 70.

Channel	I_{Rt} offset (μA)	I_{Rt} noise (nA)
Ch 1	- 30	178
Ch 2	- 32	209
Ch 3	- 34	200
Ch 4	- 40	193
Ch 5	- 40	156
Ch 6	- 45	202
B29	- 4	134

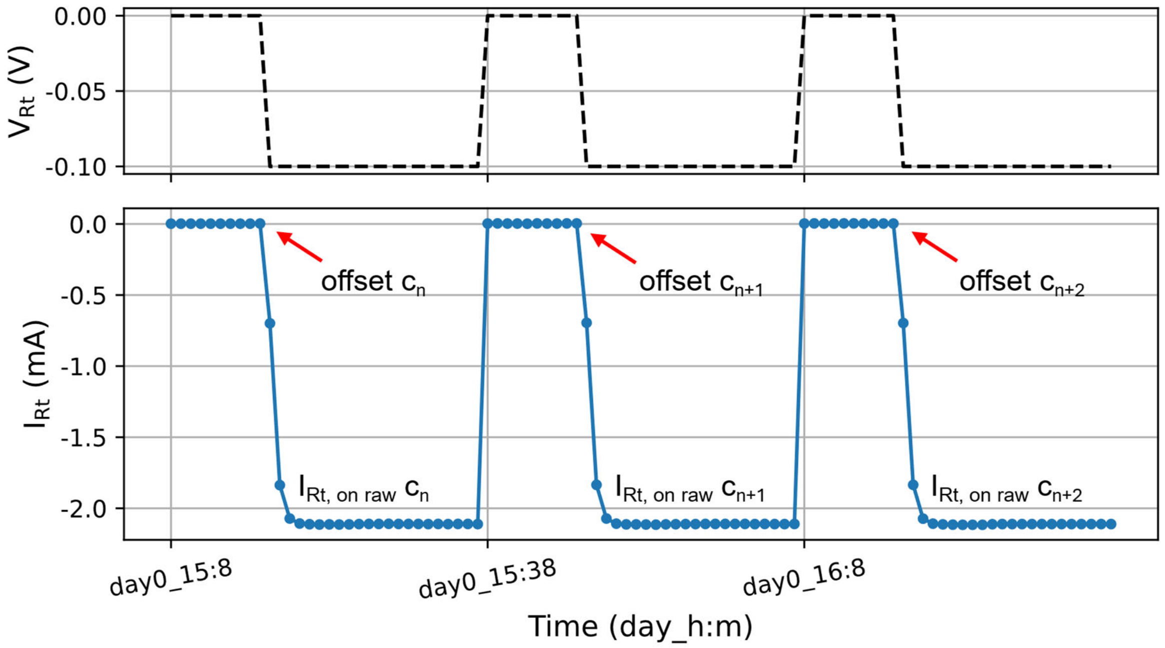

$I_{Rt,\ on}(c_x) = I_{Rt,\ on\ raw}(c_x) - \text{offset}(c_x)$

Figure S1. Illustration of the dynamic calibration method for correction of inter-channel, time-dependent offset. In post-processing, for each channel and characterization cycle ("c_x" in the figure), raw currents are corrected by subtracting the corresponding offset value acquired prior to signals application, obtaining $I_{Rt,\ on}(c_x) = I_{Rt,\ on\ raw}(c_x) - \text{offset}(c_x)$

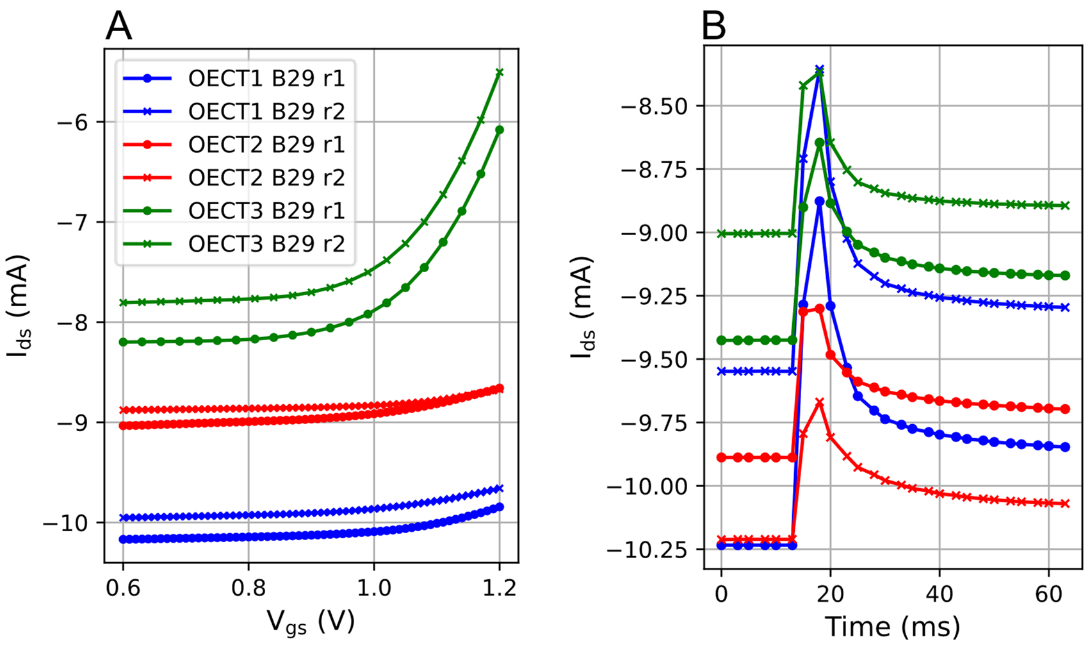

Figure S2. Characterization of three fabricated OECTs by B2912A at two different rounds. (**A**) I_{ds}–V_{gs} characterization. V_{gs} = 0.6 – 1.2 V (sweep), FSR = 20.48 mA, SPS = 128, settle time = 200 ms, step size = 10 mV. (**B**) Time response characterization. V_{gs} = 0.6 – 1.2 V (step), FSR = 20.48 mA, SPS = 860 (sampling = 2.5 ms).

Table S3. Quantitative comparison of characterization curves of three OECTs obtained by B2912A in two different rounds, as illustrated in Figure S2.

OECT	MAPE (%)	$NRMSE_{FSR}$ (%)	R^2	$g_{m, max}$ ε_r (%)	t_r ε_r (%)
OECT 1	2.2	1.1	0.991	33	11
OECT 2	1.1	0.5	0.985	31	45
OECT 3	5.6	1.2	0.999	8	24
